# Ribosome recycling induces optimal translation rate at low ribosomal availability

**DOI:** 10.1098/rsif.2014.0589

**Published:** 2014-09-06

**Authors:** E. Marshall, I. Stansfield, M. C. Romano

**Affiliations:** 1 Institute of Medical Sciences, University of Aberdeen, Foresterhill, Aberdeen AB25 2ZD, UK; 2 SUPA, Institute for Complex Systems and Mathematical Biology, King's College, University of Aberdeen, Aberdeen AB24 3UE, UK

**Keywords:** ribosome recycling, translation, totally asymmetric simple exclusion process, Rli1, ABCE1

## Abstract

During eukaryotic cellular protein synthesis, ribosomal translation is made more efficient through interaction between the two ends of the messenger RNA (mRNA). Ribosomes reaching the 3′ end of the mRNA can thus recycle and begin translation again on the same mRNA, the so-called ‘closed-loop’ model. Using a driven diffusion lattice model of translation, we study the effects of ribosome recycling on the dynamics of ribosome flow and density on the mRNA. We show that ribosome recycling induces a substantial increase in ribosome current. Furthermore, for sufficiently large values of the recycling rate, the lattice does not transition directly from low to high ribosome density, as seen in lattice models without recycling. Instead, a maximal current phase becomes accessible for much lower values of the initiation rate, and multiple phase transitions occur over a wide region of the phase plane. Crucially, we show that in the presence of ribosome recycling, mRNAs can exhibit a peak in protein production at low values of the initiation rate, beyond which translation rate decreases. This has important implications for translation of certain mRNAs, suggesting that there is an optimal concentration of ribosomes at which protein synthesis is maximal, and beyond which translational efficiency is impaired.

## 1. Introduction

**Figure 1.**
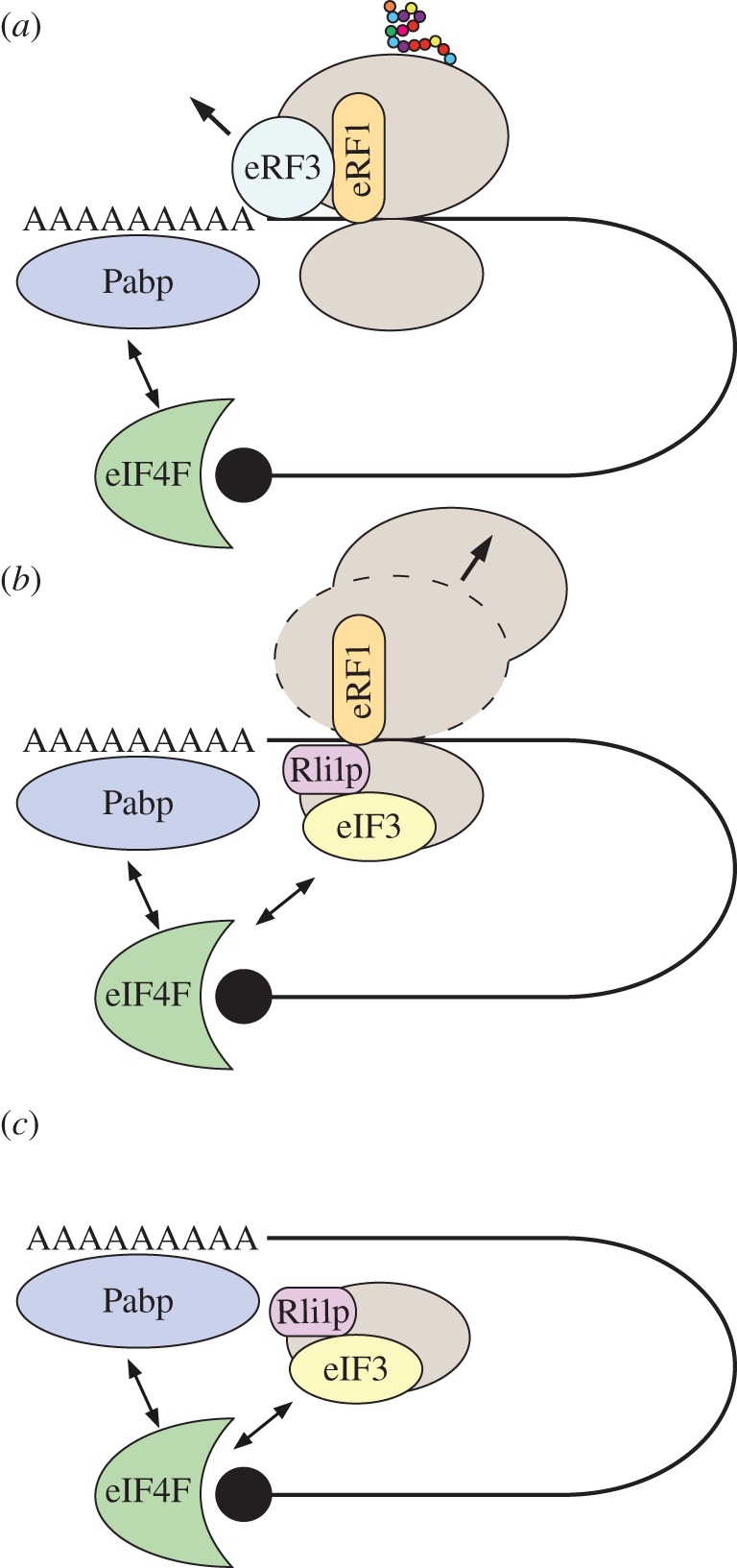
The sequence of events in ribosome recycling. (*a*) After stop-codon recognition and the 2-nucleotide toeprint shift of the ribosome, eRF3 detaches and leaves eRF1 bound to the ribosome, exposing the Rli1p binding site. (*b*) Rli1p binds and instigates detachment of the 60S subunit and release of the polypeptide chain. The Rli1p–eRF1 complex binds eIF3, which in turn recruits further initiation factors 1, 1A and 5 (not shown) to form the preinitiation complex. (*c*) The 40S subunit dissociates from the mRNA, and through direct interactions between eIF3 and the eIF4G component of the cap complex (eIF4F), the preinitiation complex can be recruited by the cap to begin translation again [5–10]. (Online version in colour.)

In this paper, we investigate the effects of recycling on eukaryotic protein synthesis by means of a mathematical model of translation that incorporates ribosome recycling. This is motivated by the ‘closed-loop’ model of translation, in which ribosomal translation is made more efficient through interactions between the two ends of the messenger RNA (mRNA) [1]. Ribosomes are large molecular machines that translate the mRNA nucleotide chain to produce the encoded protein. Translating ribosomes transit the mRNA in three-nucleotide steps. Each triplet is termed a codon, which specifies a particular amino acid [2]. Amino acids are brought to the ribosome while covalently attached to diffusing molecules called transfer RNAs (tRNAs). The mRNA ends can interact to circularize the transcript, supporting the recycling of terminating ribosomes back onto the same mRNA to commence another round of translation. Recent experimental results [3,4] suggest that this may be assisted by a highly conserved recycling factor, Rli1p (also known in mammalian systems as ABCE1), that both binds to release factors on terminating ribosomes and interacts with initiation factors to form the preinitiation complex (figure 1). Here, we show that this positive feedback mechanism has important consequences for the physics of transport of ribosomes along the mRNA, and on protein production rates under differing growth conditions.

Several previous approaches have taken into consideration the modelling of ribosome recycling [11–14], where the process has been treated mainly as passive diffusion. Essentially, in those models ribosomes are assumed to dissociate from the mRNA at the stop codon and enter the cytoplasmic pool. Owing to the proximity of the 5′ and 3′ ends of the mRNA, the local concentration of ribosomes close to the 5′ end is increased, thereby also increasing the initiation rate. However, there is experimental evidence that ribosome recycling is a more active process, with ribosomes directly passed from the 3′ to the 5′ end of the mRNA by specific translation factor interactions involving Rli1p [5–10,15,16]. Notably, depletion of Rli1p has been shown to substantially reduce gene expression in a reporter transcript in a manner that is consistent with an initiation defect [6], demonstrating its vital role in this stage of translation.

The proposed sequence of events is shown schematically in figure 1. The 3′ end of a eukaryotic mRNA contains a stretch of adenosine monophosphates, called the poly(A) tail, important for mRNA stability [17]. In turn, the 5′ end of the mRNA consists of the chemically altered guanosine (termed m^7^G, or cap), which also plays a crucial role in mRNA stability. The initiation factor eIF4G binds to the cap, and additionally, it interacts with proteins bound to the 3′ poly(A) tail of the mRNA, called poly(A)-binding proteins (Pabps), forming a ‘bridge’ between the cap and tail. When a ribosome reaches the stop codon, the release factor complex (eRF1–eRF3) binds to it, and the ribosome translocates two nucleotides downstream [18] (figure 1*a*). eRF3 then detaches from eRF1, exposing the Rli1p binding site on eRF1 [15,16] and hence allowing Rli1p to bind to eRF1. Then the 60S ribosomal subunit dissociates, leaving the ribosomal 40S subunit bound to the mRNA in a complex with eRF1 and Rli1p (figure 1*b*). Initiation factors eIF1, 1A and 3 are subsequently required in order to fully dissociate the 40S subunit from the mRNA [5], forming the preinitiation complex in concert with eIF5 in the process. The Rli1p–eRF1 complex interacts with eIF3 [6,7,9,10,16], which in turn interacts with eIF4G, as part of the mRNA cap-binding complex, in order to recruit the 40S subunit to the mRNA (figure 1*c*). There is strong experimental evidence for a direct interaction between eIF3, in particular a subunit of the protein complex called HCR1, and Rli1p. Indeed, eIF3 forms complexes with Rli1p, and to a lesser extent the release factors, even in the absence of ribosomes and mRNA [16]. Furthermore, both eIF3 and Hcr1p are present on terminating ribosomes [16]. The interaction between Rli1p and eIF3, therefore, can mediate direct ribosome progression from termination to 5′ untranslated region (UTR) scanning. Hence, a terminating ribosome can start a new round of translation on the same mRNA, in addition to cytoplasmic ribosomes which initiate de novo. As such, in our model we treat diffusive initiation as equivalent to de novo initiation and distinguish this from the active recruitment of terminating ribosomes.

In order to model the fact that ribosomes can progress directly from termination to initiation of a new round of translation, we introduce particle recycling into the totally asymmetric simple exclusion process (TASEP), a paradigmatic model of non-equilibrium statistical physics [19–21]. The TASEP has been used to model a large variety of natural and artificial transport systems [22–25]. It was originally introduced to describe translation [26], and in this particular context, it has been intensively studied and extended in the last decade [12,27–31]. In its simplest version, it consists of a one-dimensional lattice consisting of a number of *N* sites. Particles enter on the left-hand side with rate *α*, move along the lattice with rate *k* and exit on the right-hand side with rate *β*. Particles are excluded from hopping into an occupied site. The relative values of *α*, *β* and *k* give rise to characteristic mean particle densities, *ρ* (number of particles per unit lattice length), and current, *J* (number of particles passing through a site per unit time), which determine the phases that the system is in: low density (LD), if *α* < *β* and *α* < *k*/2; high density (HD), if *α* > *β* and *β* < *k*/2; shock phase (SP), if *α* = *β* and both *α*, *β* < *k*/2; or maximal current (MC), if both *α* and *β* are larger than *k*/2. The LD phase is characterized by restricted initiation and few particles making it onto the lattice. In the limit of an infinitely long lattice, the current and density are described by *J*_LD_ = *α*(1 − *α*/*k*) and *ρ*_LD_ = *α*/*k*, respectively. In the HD phase, particles are restricted from leaving the lattice, leading to queuing, with *J*_HD_ = *β*(1 − *β*/*k*) and *ρ*_HD_ = 1 − *β*/*k*. The SP is characterized by a coexistence of both LD and HD in the lattice, with the domain wall between both performing a diffusive motion [32]. Finally, the MC phase is restricted only by the internal hopping rate *k*. The particle density is optimized to allow the maximal particle current that the lattice can achieve, with 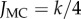 and 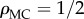 [20]. There is a phase transition of first order between the LD and HD phases (note that there is a discontinuity in the average particle density *ρ*, as well as in the first derivative of the current *J*), whereas the phase transition between LD and MC is of second order.

In the context of translation, the lattice corresponds to the mRNA, and the sites represent the codons. Ribosomes are represented by extended particles [27] given their footprint of approximately nine codons [33]. The rate *α* represents the rate at which ribosomes start translation of the mRNA, and it depends on several factors such as ribosomal and initiation factor availability, and potential secondary structures in the 5′ end of the mRNA. The rate *β* represents the rate at which ribosomes unbind the mRNA at the 3′ end, and each codon is assigned a different hopping rate *k_i_*, dependent on the availability of its cognate tRNA. The current of particles *J* corresponds to the translation rate, and the density of particles represents the average number of ribosomes bound on a mRNA divided by the mRNA length. In a genome-wide analysis of *Saccharomyces cerevisiae*, it has been shown that mRNAs can be divided into two main classes, depending on whether they exhibit a LD–HD-like (abrupt) transition as the initiation rate *α* increases, or in contrast, a LD–MC-like (smooth) transition [28,34]. This relates to the configuration of codons used by the mRNA, with *abrupt* sequences mainly presenting a slow codon, or cluster of slow codons, far from the 5′ end, whereas *smooth* sequences predominantly have slow codons close to the 5′ end or almost no slow codons at all. Importantly, the biological function of the encoded proteins significantly correlates with the type of transition given by their codon configuration. Like this, mRNAs mainly encoding regulatory and cell-cycle-related proteins are significantly enriched within the abrupt type of sequences, whereas proteins mainly involved in translation and ribosomal proteins are overrepresented in the smooth type of sequences [34].

In this paper, we introduce particle recycling into the TASEP to analyse the effects of ribosome recycling on translation. To model the fact that ribosomes can progress directly from termination to initiation mediated by Rli1p, we allow particles to either initiate again at the beginning of the lattice with rate *γ*, on the condition that the first site of the lattice is empty, or detach from the lattice with rate *β*. We show that particle recycling gives rise to a drastic increase in the current of particles, and hence the rate of protein production, as well as producing substantial changes in the phases describing the ribosome traffic. We find that for sufficiently large values of the recycling rate, the coexistence line between LD and HD phases disappears, disabling direct LD to HD transitions. Moreover, the inclusion of recycling leads to the occurrence of multiple phase transitions for a wide region of the phase diagram, and an extended MC regime on the phase plane, allowing lattices with low rates of initiation to optimize their translation rate. Remarkably, for lattices undergoing first-order phase transitions from LD to HD phases, the current of particles versus the initiation rate peaks at the point of transition, before decreasing in the HD regime. This apparently counterintuitive result suggests that for mRNAs subject to a LD to HD-like transition (e.g. those encoding mainly regulatory proteins [34]), there is an optimal cytoplasmic ribosome concentration at which the translation rate is maximal, and increasing the availability of ribosomes beyond this will in fact lead to impaired translation. Our analysis suggests that those sequences might reach the peak in their translation rate under any stress condition that limits the supply of ribosomes and, thus, the de novo initiation rate.

## 2. Active ribosome recycling model

**Figure 2.**
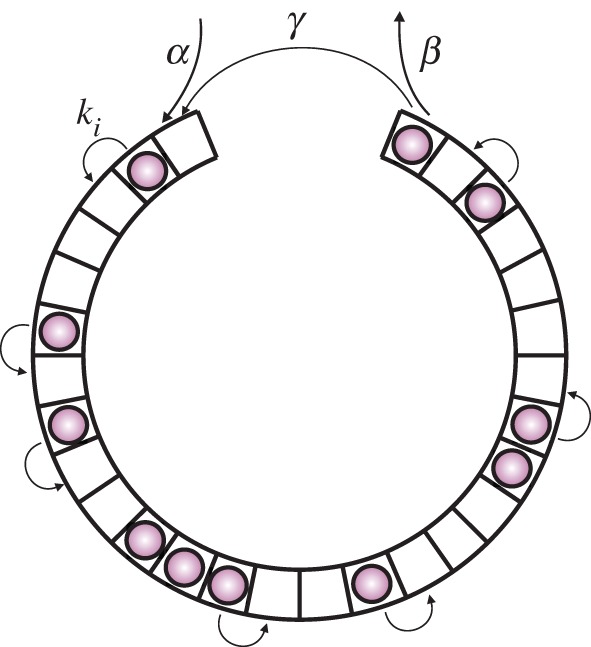
Model of translation with ribosome recycling. Ribosomes are represented by circles and the mRNA is represented by the pseudo-circular lattice. Ribosomes bind the mRNA with a rate *α*, and hop from site *i* to site *i* + 1 with rate *k_i_*. If the first site of the lattice is unoccupied, a terminating ribosome can move into this site with rate *γ* and restart translation along the mRNA. It remains the case that the terminating ribosome can also detach from the mRNA with rate *β* and re-enter the reservoir. As such, there are two potential means of ribosomes exiting the mRNA. (Online version in colour.)

As mentioned in the Introduction, terminating ribosomes can progress directly to initiation of a new round of translation on the same mRNA, mediated by the recycling factor Rli1p. Hence, we modify the original TASEP translation model by allowing particles to either exit the final site of the lattice with rate *β*, or rejoin the first site (if unoccupied), with rate *γ*, creating a superposition of open and periodic boundary conditions (figure 2). In the context of translation, site *N* − 1 corresponds to the mRNA stop codon, and site *N* takes into account the extra ribosomal translocation reaction that follows binding of the release factor complex (eRF1–eRF3) and release of the completed polypeptide [18]. Then, at site *N*, Rli1p is bound, the 60S subunit dissociates, and a complex of initiation factors binds to the 40S subunit to form the preinitiation complex (figure 1). The preinitiation complex can then be recruited to the mRNA cap with rate *γ* (if there is no steric hindrance from another complex currently initiating), or alternatively, it can be released to the cytoplasm with rate *β*. The rate *α* remains as the de novo initiation rate. As in the original TASEP, we first consider single-site particles on a homogeneous lattice, i.e. the hopping rates *k_i_* = *k* 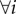 (in §5, we consider extended particles and the hopping rates *k_i_* to be proportional to their cognate tRNA abundances). We define the occupation number *n_i_*(*t*) of site *i* as being 0 if empty and 1 otherwise. Then, taking the time average 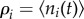, we can write the following mean field equations (i.e. we neglect correlations between sites) for the average occupation times:
2.13
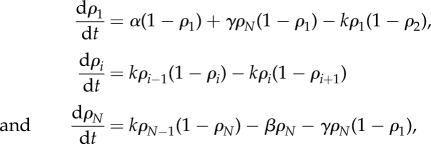

where *i* = 2, *…* ,*N* − 1. Note that the average 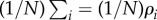 gives the mean particle density *ρ*, i.e. the average number of particles per lattice. From these equations, we derive general expressions for effective initiation and termination rates
2.1
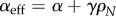

and
2.2
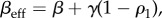

so that with *α*_eff_ and *β*_eff_, we recover the set of mean field equations for the original TASEP [20].

Then, by substituting the mean field expressions for *ρ*_1_ and *ρ_N_* [20], we derive analytical expressions for *α*_eff_ and *β*_eff_ for each of the phases, as well as for the current and mean particle density (we use calligraphic symbols to distinguish them from the original TASEP). In the following, we consider *k* = 1 for the sake of simplicity.

### 2.1. Low density

By substituting *ρ*_1_ = *α*_eff_ and 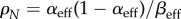 in equations (2.1) and (2.2), we obtain
2.3
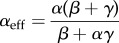

and
2.4
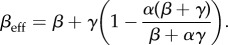



Then, knowing that 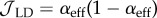 and *ρ*_LD_ = *α*_eff_, we get
2.5
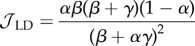

and
2.6
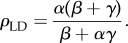



### 2.2. High density

Following the same procedure as detailed in §2.1, we substitute 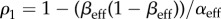 and *ρ_N_* = 1 − *β*_eff_ in equations (2.1) and (2.2) and obtain
2.7
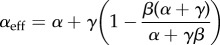

and
2.8
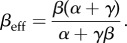



Then, by substituting these expressions in 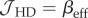 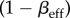 and *ρ*_HD_ = 1 − *β*_eff_, one gets
2.9
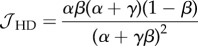

and
2.10
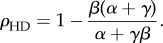



### 2.3. Maximal current

By substituting 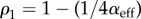 and 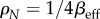 in equations (2.1) and (2.2), we obtain
2.11
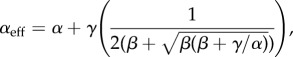

2.12
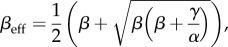

2.13
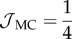

2.14
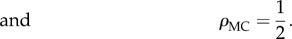



As expected from equations (2.1) and (2.2), the expressions for the effective entry and exit rates depend on the specific phases, and one recovers the original open boundaries TASEP results by setting the recycling rate *γ* = 0. The most striking effect, however, is that due to particle recycling, the current in the HD regime 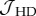 (equation (2.9)) decreases monotonically as the de novo initiation rate *α* increases, as opposed to the constant value *J*_HD_. We discuss this effect in more detail in §4.

## 3. Phase diagram

We now derive the phase diagram boundaries by substituting the obtained values for *α*_eff_ and *β*_eff_ in the conditions defining each phase (§2). The LD phase occurs if *α*_eff_ < *β*_eff_ and 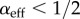. Hence, by substituting equations (2.3) and (2.4) in these inequalities, we obtain

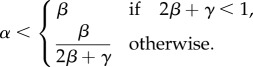



The HD phase occurs when *β*_eff_ < *α*_eff_ and 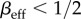. By substituting equations (2.7) and (2.8) in these inequalities, we find

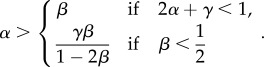



Finally, the conditions for the MC phase are 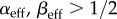. Hence, we substitute equations (2.11) and (2.12) and we obtain

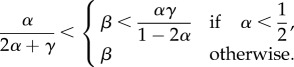



Hence, we find that the boundary lines delineating the three phases converge at the point 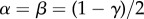, if *γ* ≤ 1.

**Figure 3.**
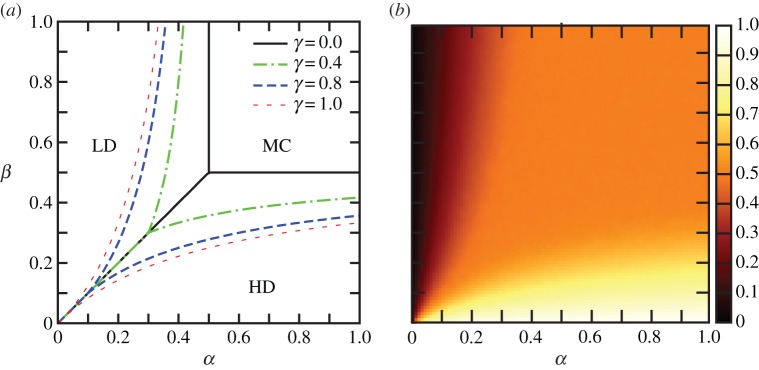
(*a*) Phase diagram for different values of the recycling rate: *γ* = 0.0 (solid black), *γ* = 0.4 (dashed-dotted green), *γ* = 0.8 (dashed blue) and *γ* = 1.0 (dotted red). The boundaries show the analytical expressions obtained by the mean field approach. (*b*) Average particle density simulated for *γ* = 0.8 and *N* = 1000, showing a good agreement with the analytical results shown in (*a*). (Online version in colour.)

The thus derived phase diagram is shown in figure 3*a*. For *γ* = 0, we recover the TASEP phase diagram, as expected (black solid line). Then, as *γ* increases, the MC phase is extended at the expense of the LD and HD phases. This has important consequences for translation, making the optimal protein production phase accessible to mRNAs for much lower values of the initiation rate *α* compared with a system without ribosome recycling. Figure 3*b* shows the results from the numerical simulations for the average density in a lattice of size *N* = 1000 sites and recycling rate *γ* = 0.8. The numerical simulations were obtained by applying the Gillespie algorithm [35], with a transient time period of 1 × 10^5^ s (during which no results were recorded in order to allow the system to reach steady state) and a total integration time of 1 × 10^6^ s. A comparison of figure 3*a*,*b* reveals a good agreement between the numerical and analytical results across the entire phase diagram.

## 4. Transitions

It is important to study how the current and density of particles, corresponding to the translation rate and ribosome density on the mRNA, respectively, change with the de novo initiation rate *α*. This allows us to predict how changes in the ribosome availability, strongly influenced by the external environment of the cell, will affect the translation rate of different mRNAs. From the expressions obtained for the boundaries between the phases in §3, one can see that if *γ* < 1, then by fixing the exit rate *β* and varying *α* systematically, we can undergo (i) a LD–HD transition if 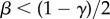, (ii) a multiple LD–MC–HD transition if 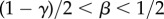, and (iii) a LD–MC transition if 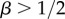. Importantly, if the recycling rate *γ* > 1, the SP or coexistence line disappears, and there is no direct LD–HD transition.

**Figure 4.**
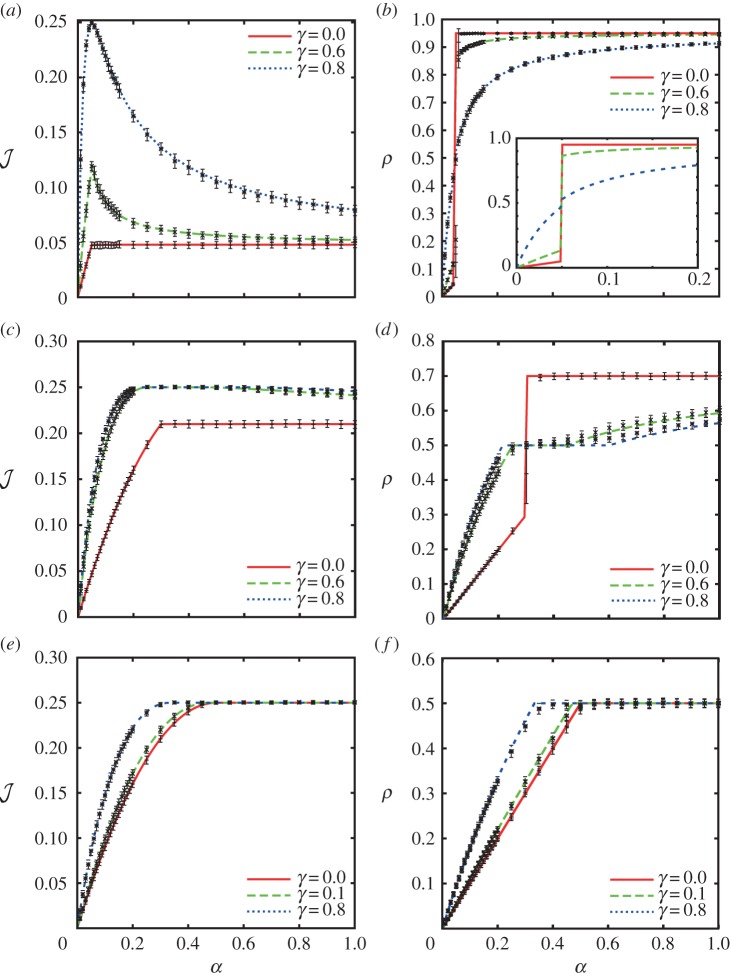
LD–HD transition: (*a*) current 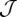 and (*b*) average density *ρ* for *β* = 0.05, *γ* = 0.0 (solid red), *γ* = 0.6 (dashed green) and *γ* = 0.8 (dotted blue). Inset (*b*): a closer view of the discontinuity in *ρ*. LD–MC–HD transition: (*c*) current 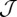 and (*d*) average density *ρ* for *β* = 0.3, *γ* = 0.0 (solid red), *γ* = 0.6 (dashed green) and *γ* = 0.8 (dotted blue). The current in (*c*) for *γ* = 0.6 and *γ* = 0.8 shows a long plateau followed by a slow decay. The transition for *γ* = 0.0 crosses the LD–HD phase boundary and accordingly, the MC phase is not entered. LD–MC transition: (*e*) current 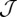 and (*f*) average density *ρ* for *β* = 0.8, *γ* = 0.0 (solid red), *γ* = 0.1 (dashed green) and *γ* = 0.8 (dotted blue). All simulations were done for a lattice of size *N* = 1000. The lines show the analytical results obtained with the mean field approach and the points show the simulation results. The bars show the standard deviation calculated in 100 windows of 5000 s. (Online version in colour.)

**Figure 5.**
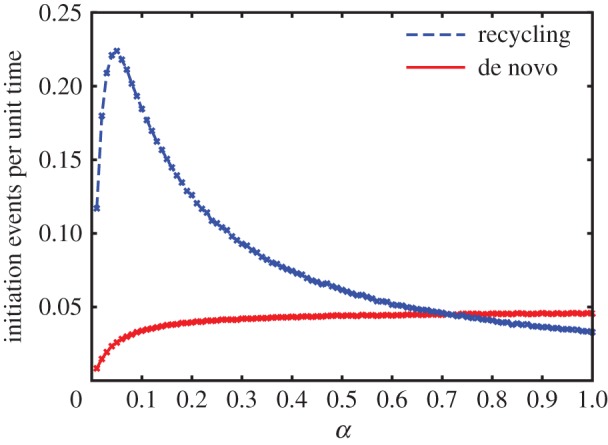
The number of initiation events per unit time by recycled ribosomes (dashed blue line) compared to de novo initiation events (solid red line). Increasing *α* leads to the percentage of de novo initiation events increasing at the expense of recycled ribosomes. Simulations were carried out for a lattice of size *N* = 1000, with *β* = 0.05 and *γ* = 0.8. A transient time period of 1 × 10^5^ s (during which no results were recorded) allowed the system to reach steady state, with a total integration time of 1 × 10^6^ s. (Online version in colour.)

We therefore consider three different values of *β*, corresponding to three possible transition scenarios in the phase diagram (LD → HD, LD → MC → HD, and LD → MC), and show how these transitions are influenced by the value of the recycling rate *γ*. Figure 4*a* shows the current, and 4*b* the average density, versus the de novo initiation rate *α* for a lattice undergoing a LD–HD transition for different values of *γ*. The current 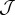 increases substantially as a consequence of particle recycling. Within the LD phase, the current increases much more rapidly than in the original TASEP (compare the dotted blue and dashed green lines with the solid red line). Remarkably, the current then shows a very pronounced maximum at the LD–HD transition, and it decreases monotonically in the HD phase, eventually converging to the value *J*_HD_ of the original TASEP when *α → ∞*. Hence, in the HD phase, higher particle availability in the reservoir for de novo initiation leads to smaller values of the current. This result, which might appear counterintuitive at first, can be understood by considering the general expression of *α*_eff_ and *β*_eff_ (equations (2.1) and (2.2)); within the HD phase, *α*_eff_ keeps increasing with *α*, leading to a substantial increase in the value of *ρ*_1_. As a consequence, the value of *β*_eff_ decreases, and since 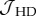 is determined by *β*_eff_, 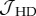 decreases with increasing de novo initiation. Figure 5 shows the number of initiation events per unit time due to recycled particles and due to de novo initiation separately versus the initiation rate *α*. The peak in initiation due to recycled particles occurs at the same value of *α* as the peak in current, i.e. at the LD–HD transition point (compare to figure 4*a*). As *α* increases beyond that point, the initiation of recycled particles decreases, until de novo initiation becomes the dominant entry mechanism. Figure 4*b* provides a different way of seeing the same effect; the average density *ρ* is higher within the LD phase (where 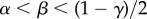, and in this simulation, when *α* < 0.1) and smaller within the HD phase compared with the non-recycling TASEP, thereby leading to a more efficient particle current. Also note that consequently, the size of the discontinuity in the average density at the LD–HD transition decreases with increasing *γ*.

Figure 4*c*,*d* show the particle current and average density, respectively, for a lattice that transitions across all three LD–MC–HD phase regimes (*β* = 0.3) for *γ* = 0.6 (dashed green line) and *γ* = 0.8 (dotted blue line). For comparison, *γ* = 0 is also shown (solid red line), which exhibits a LD–HD transition. Note that the current 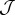 and average density *ρ* exhibit a plateau as a function of the initiation rate for the interval of *α* during which the lattice is in the MC phase. The width of this plateau is given by 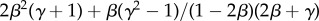, i.e. it tends to *∞* as *β →* 1/2.

In figure 4*d*, a slight disagreement between the mean-field predicted average density and the numerical results can be observed, especially as the MC phase is crossed. This is not unexpected, given the enhanced correlations within the MC phase due to recycling. This deviation, however, decreases as the lattice size is increased (see the electronic supplementary material, figure S1).

Finally, lattices undergoing a LD–MC transition show a smooth transition from the LD phase to saturation in the MC phase, as expected (figure 4*e*,*f*, for *β* = 0.8). As *γ* increases from 0.0 (solid red line) to 0.8 (dotted blue line), the transition to the MC phase occurs at lower values of *α*. This is expected from the analytical expressions obtained for the boundaries in §3, where the MC phase was seen to expand across the phase plane with increasing values of *γ*.

## 5. Application to the *Saccharomyces cerevisiae* translation system

**Figure 6.**
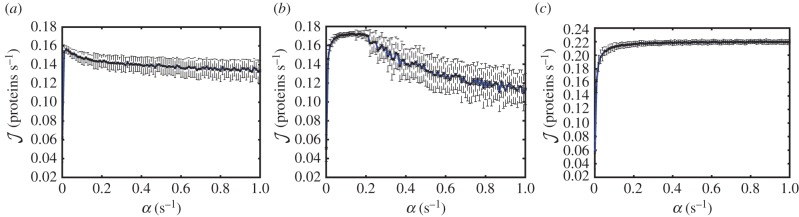
Simulations of translation rate for three representative mRNAs from *S. cerevisiae*: (*a*) *CKS1*, (*b*) *ERV46* and (*c*) *PGK1*. The bars show the standard deviation calculated in 100 windows of 2000 s. (Online version in colour.)

To assess the relevance of these results to the process of protein synthesis, we apply our model to three different representative mRNA sequences of the model organism *S. cerevisiae*. As opposed to the homogeneous lattice considered above, the hopping rates now depend on the site *i* of the lattice, each representing a codon (the derivation of these rates can be found in the electronic supplementary material, S1). These rates *k_i_* can be estimated by means of the abundances of the corresponding tRNAs [34]. Owing to the large variability in concentrations of different tRNAs, some codons are translated much faster than others; both our own work and that of others has shown that the rate of translation elongation is strongly influenced by tRNA availability [36–39], possibly in order to pause translation and permit protein folding [39,40]. There is evidence for a similar effect in other organisms including *Caenorhabditis elegans* [41], *Escherichia coli* [40,42,43] and *Mus musculus* embryos [44]. The first sequence, *CKS1*, is a cyclin-dependent protein kinase regulatory subunit, which plays a key role in the transitions between different cell cycle phases. *CKS1* presents a long stretch of slowly translated glutamine codons close to the 3′ end of the mRNA. Therefore, as the initiation rate *α* increases, it is expected to exhibit a LD–HD-like transition. To be as realistic as possible, the footprint of the ribosomes was taken into account by considering particles of size nine codons [33], and the 5′ UTR was also considered, being scanned at rate 3 s*^−^*^1^ [45]. Moreover, the hopping rate *k_N−_*_1_ = 18.03 s*^−^*^1^, corresponding to peptide release, was estimated based on the concentration of the release factors, the termination rate *β* = 0.01 s*^−^*^1^ was estimated based on *in vitro* experimental results [15], and the recycling rate *γ* = 0.8 s*^−^*^1^ was estimated from the concentration of the recycling factor Rli1p in *S. cerevisiae*, chosen as the rate determinant as it is in substantially lower abundance than eIF3 [46]. These parameters are maintained in all simulations presented. In order to avoid over-complication, we do not explicitly model every biochemical step. Rather, the rates *γ* and *α* both condense several steps that influence ribosome recruitment of the mRNA in *S. cerevisiae* such as secondary structures or availability of cap-bound initiation factors. Figure 6*a* shows how the predicted protein production rate for *CKS1* depends on the de novo initiation rate *α*. It is apparent that this sequence undergoes a LD–HD-like transition analogous to the one shown in figure 4*a*, exhibiting a maximum. Importantly, the maximum in the current occurs at the initiation rate *α* = 0.015 s*^−^*^1^, which is below our estimated average physiological initiation rate *α_ϕ_* = 0.21 s^−1^, from genome-wide simulations in combination with polysome size data from [47], analogously to [34]. Therefore, that indicates that mRNAs which undergo LD–HD-like or abrupt transitions [28,34] can reach their maximal protein synthesis rate at values of *α* lower than the physiological value. Interestingly, in the genome-wide study presented in [34], mRNAs mainly involved in transcriptional regulation and the cell cycle were significantly overrepresented in the abrupt, LD–HD-like category. Hence, the maximum in the current induced by ribosome recycling might constitute a cellular translational control mechanism that induces more efficient protein production when the availability of ribosome and initiation factors is restricted, for instance during environmental stress or nutrient restriction.

In order to eliminate the possibility that this behaviour is an artefact of the selected parameters and to show that it is due to the stretch of slow codons close to the 3′ end of *CKS1*, the stretch of slow codons was ‘mutated’ to more rapidly translated synonymous codons (i.e. coding for the same amino acid). Indeed, in this case the mutated *cks1* sequence exhibits a smooth or LD–MC-like transition (electronic supplementary material, figure S2).

Our second sequence, *ERV46* (involved in membrane fusion), has several well-spaced slow codons and shows an intermediate transition of the type LD–MC–HD, with the maximal plateau sitting well within the band of physiological values of *α* (figure 6*b*). This saturation plateau would allow these proteins to be produced at a steady rate, buffered from small changes in ribosomal availability within a certain range of *α* values. Moreover, the glycolytic enzyme *PGK1—*an mRNA sequence identified as undergoing a smooth, LD–MC-like transition [34]—was also simulated; the smooth transition is conserved in the presence of ribosome recycling (figure 6*c*). Taken together, these results indicate that recycling offers another layer of control and optimization of protein production, fine-tuning the rate of production in the face of changing ribosomal availability.

## 6. Discussion

We have proposed a new model that takes into account particle recycling in a driven diffusion lattice to study the effect of ribosome recycling in the biological process of translation. This is motivated by experimental evidence that suggests that ribosomes can pass directly from termination to the mRNA cap, via the recycling factor Rli1p. By modelling the recycling process on a homogeneous lattice with single-site particles, we have derived analytical expressions for the particle current, 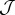, and density, *ρ*, on the lattice, analogous to the protein production rate and ribosome density on a mRNA (§2). The output of numerical simulations is in very good agreement with the analytical expressions (figures 3 and 4).

Remarkably, for lattices undergoing LD to HD transitions, the current versus the de novo initiation rate *α* exhibits a pronounced maximum at the interface between both phases. Furthermore, within the HD phase, the current decreases with increasing initiation rate. This result seems counterintuitive at first, because it means that the higher the availability of ribosomes, the smaller the translation rate. However, this effect can be understood by noting that as the de novo initiation rate *α* increases, the proportion of recycling initiation events vanishes, eventually converging to the regime in the absence of recycling, namely, the original TASEP (figure 5). Furthermore, we have shown that, apart from the expected increase in the current of the system, the phase diagram changes substantially: the MC phase is considerably extended, so that it is accessible from much lower values of the initiation rate; multiple phase transitions are possible in a wide region of the phase plane; and the coexistence line between the LD and HD phases vanishes if the recycling rate becomes sufficiently large (*γ* ≥ 1 in the homogeneous case).

The model was applied to real sequences from the budding yeast *S. cerevisiae* and the three main types of phase transition were observed: LD–HD-like, LD–MC–HD-like and LD–MC-like. Following the findings in [34] that regulatory proteins are overrepresented in the abrupt transition category, we simulated the sequence for *CKS1*, involved in regulation of cell cycle transitions, and with a stretch of slowly translated glutamine codons towards the end of the transcript acting to reduce the effective termination rate. The result of this simulation confirmed *CKS1* as undergoing an abrupt transition from the LD to HD phase. Furthermore, as seen in the analytical plots of 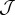 in §2, the current was observed to peak at the point of phase transition, followed by a subsequent decrease in the HD phase. The peak occurs at a value of the initiation rate *α* that is lower than the predicted physiological value. This has important consequences for protein synthesis, suggesting that ribosome recycling provides the cell with an additional control mechanism to optimize production of regulatory proteins upon environmental stress or nutrient depletion, when ribosomes are depleted.

The sequence for *ERV46*, involved in membrane fusion, demonstrated a LD–MC–HD-like transition. There is a defined plateau in the current, 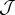, indicating that the protein synthesis rate is saturated for a range of de novo initiation rates. This saturation plateau could buffer the rate of protein production from small changes in ribosomal availability, ensuring a steady supply of *ERV46* within a defined band of *α* values. Taking these results together, they signal that ribosome recycling could offer the cell a further layer of regulation of gene expression, with the ability to fine-tune protein production in synergy with cellular ribosomal concentration.

Molecular biology experiments involving mutants of the recycling factor Rli1p in *S. cerevisiae* are being designed at the moment to validate the model's predictions. It has been shown that depletion of this factor substantially reduces expression of a reporter gene [6] and overexpression rescues the growth rate in *hcr1*Δ strains [16]; we are combining the mutation of Rli1p with changes in the de novo initiation rate to investigate the effect on protein synthesis and ribosome density on the mRNA. Additional work is planned to study the effects of competition for ribosomes among a population of ribosome-recycling mRNAs, as well as effects of the ribosome mechano-chemical cycle [29] on recycling.

## Supplementary Material

Ribosome recycling induces optimal translation rate at low ribosomal availability: Supplementary material

## References

[1] Amrani N Ghosh S Mangus DA Jacobson A 2008 Translation factors promote the formation of two states of the closed-loop mRNP. Nature 453, 1276–1280. (10.1038/nature06974) 18496529 PMC2587346

[2] Alberts B Johnson A Lewis J Raff M Roberts K Walter P 2008 Molecular biology of the cell, 5th edn New York, NY: Garland Science.

[3] Barthelme D Dinkelaker S Albers S-V Londei P Ermler U Tampé R 2011 Ribosome recycling depends on a mechanistic link between the FeS cluster domain and a conformational switch of the twin-ATPase ABCE1. Proc. Natl Acad. Sci. USA 108, 3228–3233. (10.1073/pnas.1015953108) 21292982 PMC3044390

[4] Becker T 2012 Structural basis of highly conserved ribosome recycling in eukaryotes and archaea. Nature 482, 501–506. (10.1038/nature10829) 22358840 PMC6878762

[5] Pisarev AV Skabkin MA Pisareva VP Skabkina OV Rakotondrafara AM Hentze MW Hellen CUT Pestova TV 2010 The role of ABCE1 in eukaryotic posttermination ribosomal recycling. Mol. Cell 37, 196–210. (10.1016/j.molcel.2009.12.034) 20122402 PMC2951834

[6] Dong J Lai R Nielsen K Fekete CA Qiu H Hinnebusch AG 2004 The essential ATP-binding cassette protein RLI1 functions in translation by promoting preinitiation complex assembly. J. Biol. Chem. 279, 42 157–42 168. (10.1074/jbc.M404502200) 15277527

[7] Fraser CS Lee JY Mayeur GL Bushell M Doudna JA Hershey JWB 2004 The j-subunit of human translation initiation factor eIF3 is required for the stable binding of eIF3 and its subcomplexes to 40S ribosomal subunits *in vitro**.* J. Biol. Chem. 279, 8946–8956. (10.1074/jbc.M312745200) 14688252

[8] Franckenberg S Becker T Beckmann R 2012 Structural view on recycling of archaeal and eukaryotic ribosomes after canonical termination and ribosome rescue*.* Curr. Opin. Struct. Biol. 22, 786–796. (10.1016/j.sbi.2012.08.002) 23031510

[9] Yarunin A Panse VG Petfalski E Dez C Tollervey D Hurt EC 2005 Functional link between ribosome formation and biogenesis of iron-sulfur proteins. EMBO J. 24, 580–588. (10.1038/sj.emboj.7600540) 15660135 PMC548649

[10] Kispal G 2005 Biogenesis of cytosolic ribosomes requires the essential iron-sulphur protein Rli1p and mitochondria. EMBO J. 24, 589–598. (10.1038/sj.emboj.7600541) 15660134 PMC548650

[11] Gilchrist MA Wagner A 2006 A model of protein translation including codon bias, nonsense errors and ribosome recycling. J. Theor. Biol. 239, 417–434. (10.1016/j.jtbi.2005.08.007) 16171830

[12] Chou T 2003 Ribosome recycling, diffusion, and mRNA loop formation in translational regulation. Biophys. J. 85, 755–773. (10.1016/S0006-3495(03)74518-4) 12885626 PMC1303200

[13] Sharma AK Chowdhury D 2011 Stochastic theory of protein synthesis and polysome: ribosome profile on a single mRNA transcript. J. Theor. Biol. 289, 36–46. (10.1016/j.jtbi.2011.08.023) 21888920

[14] Margaliot M Tuller T 2013 Ribosome flow model with positive feedback. J. R. Soc. Interface 10, 20130267 (10.1098/rsif.2013.0267) 23720534 PMC4043157

[15] Shoemaker CJ Green R 2011 Kinetic analysis reveals the ordered coupling of translation termination and ribosome recycling in yeast. Proc. Natl Acad. Sci. 108, E1392–E1398. (10.1073/pnas.1113956108) 22143755 PMC3251084

[16] Beznosková P Cuchalová L Wagner S Shoemaker CJ Gunišová S von der Haar T Valášek LS 2013 Translation initiation factors eIF3 and HCR1 control translation termination and stop codon read-through in yeast cells. PLoS Genet. 9, e1003962 (10.1371/journal.pgen.1003962) 24278036 PMC3836723

[17] Bernstein P Peltz SW Ross J 1989 The poly(A)-poly(A)-binding protein complex is a major determinant of mRNA stability in vitro. Mol. Cell Biol. 9, 659–670. (10.1128/MCB.9.2.659) 2565532 PMC362643

[18] Kryuchkova P Grishin A Eliseev B Karyagina A Frolova L Alkalaeva E 2013 Two-step model of stop codon recognition by eukaryotic release factor eRF1. Nucleic Acids Res. 41, 4573–4586. (10.1093/nar/gkt113) 23435318 PMC3632111

[19] Spitzer F 1970 Interaction of Markov processes. Adv. Math. 5, 246–290. (10.1007/978-1-4612-0459-6_5)

[20] Derrida B Domany E Mukamel D 1992 An exact solution of a one-dimensional asymmetric exclusion model with open boundaries. J. Stat. Phys. 69, 667–687. (10.1088/0305-4470/26/7/011)

[21] Chou T Mallick K Zia RKP 2011 Non-equilibrium statistical mechanics: from a paradigmatic model to biological transport. Rep. Prog. Phys. 74, 116601 (10.1088/0034-4885/74/11/116601)

[22] Hilhorst HJ Appert-Rolland C 2012 A multi-lane TASEP model for crossing pedestrian traffic flows. J. Stat. Mech. 2012, P06009 (10.1088/1742-5468/2012/06/P06009)

[23] Popkov V Santen L Schadschneider A Schütz GM 2001 Empirical evidence for a boundary-induced nonequilibrium phase transition. J. Phys. A Math. Gen. 34, L45–L52. (10.1088/0305-4470/34/6/103)

[24] Neri I Kern N Parmeggiani A 2011 Totally asymmetric simple exclusion process on networks. Phys. Rev. Lett. 107, 068702 (10.1103/PhysRevLett.107.068702) 21902376

[25] Chowdhury D Schadschneider A Nishinari K 2005 Physics of transport and traffic phenomena in biology: from molecular motors and cells to organisms. Phys. Life Rev. 2, 318–352. (10.1016/j.plrev.2005.09.001)

[26] MacDonald CT Gibbs JH Pipkin AC 1968 Kinetics of biopolymerization on nucleic acid templates. Biopolymers 6, 1–25. (10.1002/bip.1968.360060102) 5641411

[27] Shaw LB Zia RKP Lee KH 2003 Totally asymmetric exclusion process with extended objects: a model for protein synthesis. Phys. Rev. E 68, 021910 (10.1103/PhysRevE.68.021910) 14525009

[28] Romano MC Thiel M Stansfield I Grebogi C 2009 Queuing phase transition: theory of translation. Phys. Rev. Lett. 102, 198104 (10.1103/PhysRevLett.102.198104) 19519001 PMC3639427

[29] Ciandrini L Stansfield I Romano MC 2010 Role of the particle's stepping cycle in an asymmetric exclusion process: a model of mRNA translation. Phys. Rev. E 81, 051904 (10.1103/PhysRevE.81.051904) PMC3639468 20866258

[30] Brackley CA Romano MC Thiel M 2011 The dynamics of supply and demand in mRNA translation. PLoS Comput. Biol. 7, e1002203 (10.1371/journal.pcbi.1002203) 22022250 PMC3192816

[31] Zia RKP Dong JJ Schmittmann B 2011 Modeling translation in protein synthesis with TASEP: a tutorial and recent developments. J. Stat. Phys. 144, 405–428. (10.1007/s10955-011-0183-1)

[32] Kolomeisky AB Schütz GM Kolomeisky EB Straley JP 1998 Phase diagram of one-dimensional driven lattice gases with open boundaries. J. Phys. A Math. Gen. 31, 6911–6919. (10.1088/0305-4470/31/33/003)

[33] Ingolia NT Ghaemmaghami S Newman JRS Weissman JS 2009 Genome-wide analysis *in vivo* of translation with nucleotide resolution using ribosome profiling. Science 324, 218–223. (10.1126/science.1168978) 19213877 PMC2746483

[34] Ciandrini L Stansfield I Romano MC 2013 Ribosome traffic on mRNAs maps to gene ontology: genome-wide quantification of translation initiation rates and polysome size regulation. PLoS Comput. Biol. 9, e1002866 (10.1371/journal.pcbi.1002866) 23382661 PMC3561044

[35] Gillespie DT 1977 Exact stochastic simulation of coupled chemical reactions. J. Phys. Chem. 81, 2340–2361. (10.1021/j100540a008)

[36] Kemp AJ Betney R Ciandrini L Schwenger ACM Romano MC Stansfield I 2013 A yeast tRNA mutant that causes pseudohyphal growth exhibits reduced rates of CAG codon translation. Mol. Microbiol. 87, 284–300. (10.1111/mmi.12096) 23146061 PMC3664417

[37] Tuller T 2010 An evolutionarily conserved mechanism for controlling the efficiency of protein translation. Cell 141, 344–354. (10.1016/j.cell.2010.03.031) 20403328

[38] Percudani R Pavesi A Ottonello S 1997 Transfer RNA gene redundancy and translational selection in *Saccharomyces cerevisiae*. J. Mol. Biol. 268, 322–330. (10.1006/jmbi.1997.0942) 9159473

[39] Pechmann S Frydman J 2013 Evolutionary conservation of codon optimality reveals hidden signatures of cotranslational folding. Nat. Struct. Mol. Biol. 20, 237–243. (10.1038/nsmb.2466) 23262490 PMC3565066

[40] Komar A Lesnik T Reiss C 1999 Synonymous codon substitutions affect ribosome traffic and protein folding during in vitro translation. FEBS Lett. 462, 387–391. (10.1016/S0014-5793(99)01566-5) 10622731

[41] Stadler M Fire A 2011 Wobble base-pairing slows *in vivo* translation elongation in metazoans. RNA 17, 2063–2073. (10.1261/rna.02890211) 22045228 PMC3222120

[42] Varenne S Buc J Lloubes R Lazdunski C 1984 Translation is a non-uniform process. Effect of tRNA availability on the rate of elongation of nascent polypeptide chains. J. Mol. Biol. 180, 549–576. (10.1016/0022-2836(84)90027-5) 6084718

[43] Angov E Hillier CJ Kincaid RL Lyon JA 2008 Heterologous protein expression is enhanced by harmonizing the codon usage frequencies of the target gene with those of the expression host. PLoS ONE 3, e2189 (10.1371/journal.pone.0002189) 18478103 PMC2364656

[44] Dana A Tuller T 2012 Determinants of translation elongation speed and ribosomal profiling biases in mouse embryonic stem cells. PLoS Comput. Biol. 8, e1002755 (10.1371/journal.pcbi.1002755) 23133360 PMC3486846

[45] Nagalakshmi U Wang Z Waern K Shou C Raha D Gerstein M Snyder M 2008 The transcriptional landscape of the yeast genome defined by RNA sequencing. Science 320, 1344–1349. (10.1126/science.1158441) 18451266 PMC2951732

[46] Ghaemmaghami S Huh W-K Bower K Howson RW Belle A Dephoure N O'Shea EK Weissman JS 2003 Global analysis of protein expression in yeast. Nature 425, 737–741. (10.1038/nature02046) 14562106

[47] MacKay VL 2004 Gene expression analyzed by high-resolution state array analysis and quantitative proteomics. Mol. Cell. Proteomics 3, 478–498. (10.1074/mcp.M300129-MCP200) 14766929

