# Supplementary material for: Ribosome recycling induces optimal translation rate at low ribosomal availability
Source: J R Soc Interface. 2014 Sep 6;11(98):20140589. doi: 10.1098/rsif.2014.0589 (PMC4233708; doi:10.1098/rsif.2014.0589)
Supplement: Ribosome recycling induces optimal translation rate at low ribosomal availability: Supplementary material [file rsif20140589supp1.pdf]

Ribosome recycling induces optimal translation rate at low ribosomal availability: Supplementary material

E. Marshall*[1,2], I. Stansfield[1], and M. C. Romano†[1,2]

[1]*Institute of Medical Sciences, Foresterhill, University of Aberdeen, Aberdeen, AB25 2ZD, United Kingdom*
[2]*SUPA, Institute for Complex Systems and Mathematical Biology, King's College, University of Aberdeen, Aberdeen, AB24 3UE, United Kingdom*

S1. Derivation of *k*-values and 5' UTR lengths

The rate of movement of ribosomes from codon i to $i+1$, k_i, can be estimated from the abundance of the cognate tRNA at codon i, assumed to be proportional to the tRNA gene copy number (GCN) [1]. A first estimate of the hopping rates is then given by the following formula:

$$k_j = r\frac{GCN_j}{\sum_{j=1}^{41} GCN_j}. \quad (1)$$

Here *r* is a proportionality constant that averages the rate of elongation, $\langle k_i \rangle$, to 10 amino acids/s, as per Gilchrist & Wagner [2].

In its simplest form this would give rise to a total of 41 rates corresponding to each of the 41 tRNAs. However, we have also accounted for Crick's wobble base-pairing, where those codons translated using the G-U wobble have their k_i reduced by 39% compared to the Watson-Crick base pairing of G-C. Similarly those codons with the I-C and I-A wobble have been shown to have their k_i reduced by 36% compared to I-U [2]. Factoring this into our model results in a total of 61 elongation rates.

We calculate the proportionality constant *r* using

$$\langle k_i \rangle = \sum_{j=1}^{61} k_i \frac{n_i}{n}, \quad (2)$$

where n_i is the total number of codons of type *i* across all mRNA in the cell, and $n = \sum_{i=1}^{61} n_i$. By equating this to 10s^{-1} we can calculate *r*. The so-calculated hopping rates k_i are listed in Table S1.

We have also taken the 5' UTR scanning process into account, where the 40S ribosomal subunit scans along the mRNA until it identifies a start codon. The table of 5' UTR lengths calculated by Tuller, Ruppin & Kupiec was used to assign lengths to sequences where these are known [3]. Sequences modelled here with an undetermined 5' UTR length were assigned the median value of 54 nucleotides.

*emarshall@abdn.ac.uk
†m.romano@abdn.ac.uk

Table S1. Amino acids and their corresponding anti-codons, codons, and translation rates (k-values). The k-values were calculated as described in Section S1.

Amino Acid	Anti-Codon	Codon	Translation Rate
ala	IGC	GCU	12.39
		GCC	7.93
	UGC	GCA	5.63
		GCG	3.44
arg	ICG	CGU	6.76
		CGC	4.33
		CGA	4.33
	CCG	CGG	1.13
	UCU	AGA	12.39
	CCU	AGG	1.13
asn	GUU	AAU	6.87
		AAC	11.27
asp	GUC	GAU	10.31
		GAC	16.90
cys	GCA	UGU	2.75
		UGC	4.51
gln	UUG	CAA	10.14
	CUG	CAG	1.13
	UUC	GAA	15.77
	CUC	GAG	2.25
gly	GCC	GGU	11.00
		GGC	18.03
	UCC	GGA	3.38
	CCC	GGG	2.25
his	GUG	CAU	4.81
		CAC	7.89
ile	IAU	AUU	14.65
		AUC	9.37
	UAU	AUA	2.25
leu	UAA	UUA	7.89
	CAA	UUG	11.27
	GAG	CUU	0.69
		CUC	1.13
	UAG	CUA	3.38
		CUG	2.06
lys	UUU	AAA	7.89
	CUU	AAG	15.77
met	CAU	AUG	5.63
phe	GAA	UUU	6.87
		UUC	11.27
pro	IGG	CCU	2.25
		CCC	1.44
	UGG	CCA	11.27
		CCG	6.87
ser	IGA	UCU	12.39

Continued on next page

Table 1 – continued from previous page

Amino Acid	Anti-Codon	Codon	Translation Rate
		UCC	7.93
	UGA	UCA	3.38
	CGA	UCG	1.13
	GCU	AGU	2.75
		AGC	4.51
thr	IGU	ACU	12.39
		ACC	7.93
	UGU	ACA	4.51
	CGU	ACG	1.13
trp	CCA	UGG	6.76
tyr	GUA	UAU	5.50
		UAC	9.01
val	IAC	GUU	15.77
		GUC	10.1
	UAC	GUA	2.25
	CAC	GUG	2.25

S2. Increasing the lattice length improves mean field agreement

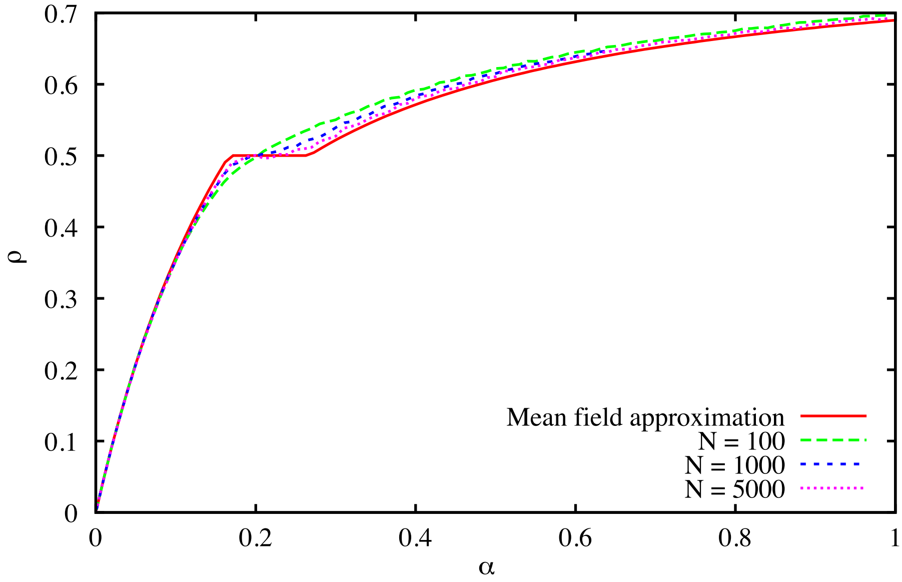

Figure 1: The mean field approach is less accurate when the MC phase is crossed, due to enhanced long-range correlations on the lattice under recycling. The disagreement, evident in graphs of ρ vs the initiation rate, when undergoing LD-MC-HD transitions, can be improved by increasing the lattice size. Here $\beta = 0.2$ and $\gamma = 0.8$. The total integration time was $1.5 \cdot 10^6$ s, including a transient period of $5 \cdot 10^5$ s. (Online version in colour.)

S3. Mutating the *CKS1* sequence changes the type of phase transition

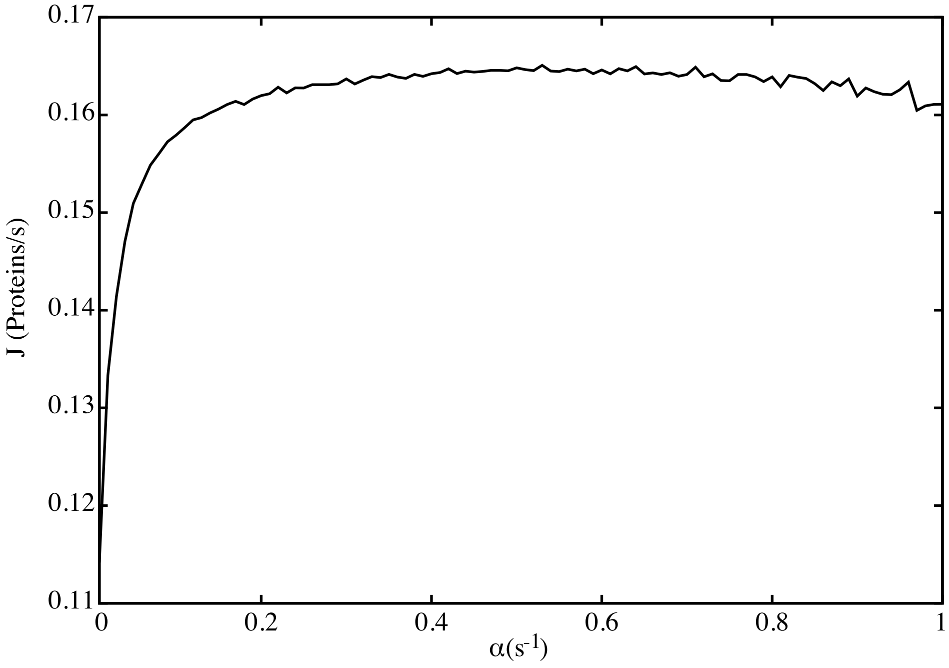

Figure 2: Mutating the cluster of slow glutamine codons found at the end of the *CKS1* mRNA leads to a change from the abrupt LD-HD-like transition, to a smooth LD-MC-like transition. This confirms that the LD-HD-like transition behaviour is a product of the cluster of slow codons at the 3' end of the mRNA.

References

[1] Percudani R, Pavesi A, Ottonello S. 1997 Transfer RNA gene redundancy and translational selection in *Saccharomyces cerevisiae*. *J. Mol. Biol.* **268**, 322-330. (doi:10.1006/jmbi.1997.0942)

[2] Gilchrist MA, Wagner A. 2006 A model of protein translation including codon bias, nonsense errors and ribosome recycling. *J. Theor. Biol.* **239**, 417-434. (doi:10.1016/j.jtbi.2005.08.007)

[3] Tuller T, Ruppin E, Kupiec M. 2009 Properties of untranslated regions of the *S. cerevisiae* genome. *BMC Genomics* **10**, 391-402. (doi:10.1186/1471-2164-10-391)
